# Immunocytochemistry Profile of Benign Thyroid Nodules Not Responding to Thermal Ablation: A Retrospective Study

**DOI:** 10.1155/2023/7951942

**Published:** 2023-04-11

**Authors:** Stella Bernardi, Silvia Taccogna, Martina D'Angelo, Fabiola Giudici, Giovanni Mauri, Bruno Raggiunti, Doris Tina, Fabrizio Zanconati, Enrico Papini, Roberto Negro

**Affiliations:** ^1^Department of Medical Surgical and Health Sciences, University of Trieste, Cattinara Teaching Hospital, Strada di Fiume, Trieste 34149, Italy; ^2^SS Endocrinologia UCO Medicina Clinica, Cattinara Teaching Hospital, Strada di Fiume, Trieste 34149, Italy; ^3^Pathology Unit, Regina Apostolorum Hospital, Albano Laziale (RM) 00041, Italy; ^4^Bureau Biostatistique et Epidémiologie, Gustave-Roussy Institute, Rue Eduard Vaillant, Villejuif 94805, France; ^5^IRCCS European Institute of Oncology, Milano 20141, Italy; ^6^Department of Oncology, University of Milan, Milan 20100, Italy; ^7^UOC Malattie Endocrine e Diabetologia, PO di Atri, ASL Teramo, Teramo, Italy; ^8^UCO Anatomia Patologica, Cattinara Teaching Hospital, Strada di Fiume, Trieste 34149, Italy; ^9^Endocrinology Department, Regina Apostolorum Hospital, Albano Laziale (RM) 00041, Italy; ^10^UO Endocrinologia, “V. Fazzi” Hospital, Lecce 73100, Italy

## Abstract

**Purpose:**

Thermal ablations (TA) are gaining ground as alternative options to conventional therapies for symptomatic benign thyroid nodules. Little is known about the impact of nodule biology on the outcomes of TA. The aim of our study was to evaluate the baseline immunocytochemistry profile of thyroid nodules that were poorly responsive to TA in order to identify potential predictors of the treatment response.

**Methods:**

From a cohort of 406 patients with benign thyroid nodules treated with TA and followed for 5 years, we retrospectively selected two groups of patients: NONRESPONDERS (patients who did not respond to TA and were later surgically treated) and RESPONDERS (patients who responded to TA). The fine-needle aspiration cytology (FNAC) slides obtained before TA were stained for Galectin-3, HBME-1, CK-19, and Ki-67.

**Results:**

Benign nodules of NONRESPONDERS (*n* = 19) did not express CK-19 (*p* = 0.03), as compared to RESPONDERS (*n* = 26). We combined the absence of CK-19 and the presence of Ki-67 to obtain a composite biomarker of resistance to TA, which discriminated between likelihood of retreatment and no retreatment with an AUC of 0.68 (95%CI: 0.55-0.81) and a sensitivity, specificity, PPV, and NPV of 29%, 91%, 71%, and 64%, respectively.

**Conclusion:**

In benign thyroid nodules, the absence of CK-19 was associated with resistance to TA, while the presence of CK-19 was predictive of response to TA. If confirmed, this finding could provide rapid and inexpensive information about the potential outcome of TA on benign thyroid nodules. In addition, as CK-19 can be expressed in adenomatous hyperplasia, it could be speculated that these nodules, rather than follicular adenomas, might be better candidates for TA.

## 1. Introduction

Thermal ablations (TA) with laser (LTA), radiofrequency (RFA), or microwaves (MWA) are gaining ground as an alternative therapeutic option to surgery for the management of symptomatic benign thyroid nodules [1, 2]. TA are specifically suited for single, nonfunctioning, benign thyroid nodules with a mixed or solid structure that cause local symptoms or cosmetic concerns [3]. By contrast, ethanol ablation is the first-line minimally-invasive technique for cystic nodules, while radioiodine and surgery remain the preferred therapeutic option for autonomously functioning thyroid nodules [1, 2, 4].

In single, nonfunctioning, benign thyroid nodules, TA result in a clinically effective decrease of the size of the nodule, which usually shrinks by 60–80% one year after treatment and is associated with improvement or disappearance of local symptoms and cosmetic concerns [5]. Recent studies demonstrate that several variables concerning the nodule characteristics and the modalities of treatment might influence the outcome of the procedure. For instance, the baseline nodule volume [6, 7] and its composition [6, 8], as well as the energy delivered [8–10], have been independently associated with the final volume reduction. We have recently reported that young age, a large baseline volume, and a low amount of energy delivered are also associated with the risk of regrowth and the need for further treatment [11]. In addition, the initial ablation ratio, which is a semiquantitative index that measures the amount of tissue ablation 1–3 months after the procedure [12], as well as the residual vital ratio, can predict the volume reduction and/or the opportunity of retreatment [12–14].

A few immunocytochemical markers have been investigated and used for the differential diagnosis between benign and malignant thyroid nodules with indeterminate cytologic results on fine-needle aspiration cytology (FNAC) samples [15, 16]. These immunocytochemical markers include galectin-3, HBME-1 (Hector Battifora Mesothelial cell) [17, 18], cytokeratin (CK)-19, as well as the Ki-67 proliferation index. Galectin-3 is a member of lectin family, which plays a major role in cell growth and differentiation, apoptosis, malignant transformation, and metastasis [19]. HBME-1 is a known marker of mesothelial cells, and in a recent meta-analysis, galectin-3 and HBME-1 had the highest accuracy in discriminating benign from malignant thyroid nodules [20]. CK-19 is a low-molecular-weight cytokeratin found in basal layers of stratified epithelium and simple epithelia, which is upregulated in neoplastic transformation, particularly in the case of papillary thyroid carcinomas. As for the marker of proliferation Ki-67, which is predominantly expressed during the *S*, *G*_2_, and *M* phases of the cell cycle, it can help identify patients with moderate or high risk of thyroid carcinoma, where its expression is usually in the range of 5–10% (moderate risk) or 10–30% (high risk) [21, 22]. By contrast, in follicular adenomas, Ki-67 expression is generally <3% [21].

Presently, little is known on the role of these immunocytochemical markers in the growth rate of cytologically benign thyroid nodules and the impact of nodule biology on the outcome of TA procedures [23, 24]. Based on these premises, the aim of our study was to assess the immunocytochemical profile of thyroid nodules that either responded or not responded to TA in order to identify potential new predictors of treatment outcome.

## 2. Materials and Methods

### 2.1. Study Design and Population

This is a retrospective study aiming to evaluate if benign thyroid nodules that failed to respond to TA had a different baseline immunocytochemical profile as compared to those that showed a favorable outcome after the procedure. The outcomes of the TA procedure were defined in accordance with the standardization of terminology and reporting criteria for image-guided thyroid ablation [25]. So, technique efficacy was defined as a volumetric reduction >50% of the initial nodule volume 1 year after treatment; regrowth was defined as a >50% increase compared to the previous smallest volume at US examination.

Two groups of patients, namely NONRESPONDERS (patients who did not respond to TA and were later surgically treated) and RESPONDERS (patients who responded to TA and were not retreated), were retrospectively selected by the propensity score matching method from the data set of a recent multicenter study published by our group [11]. In that study, we had collected the data of 406 patients with benign thyroid nodules who were treated with TA between 2014 and 2019 in 8 Italian Centers and then were followed for at least 5 consecutive years. Among them, 46 patients did not respond to TA and were surgically retreated (NONRESPONDERS), whereas 360 patients responded satisfactorily to TA and did not require any further retreatment (RESPONDERS). Other details can be found in the original article [11].

Propensity score matching (PSM) (1 : 1) was performed to balance the selection bias and potential confounding factors between NONRESPONDERS and RESPONDERS and to find appropriate controls to the 46 NONRESPONDERS. PSM was calculated based on baseline characteristics including age, sex, nodule volume, nodule structure, nodule function, and technique (RFA vs. LA). Energy delivered was not included in the PSM because this covariate is correlated to the procedure (RFA vs. LA) [11], and the inclusion of correlated covariates might affect the efficiency of the estimators and then of the PSM [26]. For this reason, we decided to introduce the technique (RFA vs. LA) and not energy in the PSM. Finally, 32 matched pairs were generated (32 NONRESPONDERS vs. 32 RESPONDERS patients = 64 patients).

This study was conducted in accordance with the declaration of Helsinki, and its protocol was approved by the Institutional Review Board of ASUGI/ARCS (268-2019 FYTNAB).

### 2.2. Immunocytochemistry

Based on the PSM results, we retrospectively searched the FNAC slides of the 64 patients selected and managed to collect the slides of 50 patients out of the 64. They included specimens of 24 NONRESPONDERS and 26 RESPONDERS from 3 centers (Lecce, Teramo, and Trieste). All FNAC were performed before the procedure (before 2014), and the cytology was diagnostic for benign thyroid nodule (Bethesda II diagnostic category), which is a prerequisite for treating with TA [1, 2, 5].

Immunocytochemistry was performed on decolorized Papanicolaou-stained smears after fixation with 20% phosphate-buffered formalin solution for 30 min and antigen retrieval at 95°C for 30 min with an activation solution specific to our equipment. FNAC slides were incubated with antibodies for Galectin-3, HBME-1, CK-19, and Ki-67 on an automated immunostainer using EnVision DAB Flex (Agilent Technologies, Denmark).

Based on the material that was collected (a few slides for each patient), 45 slides were stained for galectin-3 and 45 for CK-19 (22 NONRESPONDERS and 23 RESPONDERS), while 50 slides were stained for HBME-1 and 50 slides were stained for Ki-67 (24 NONRESPONDERS and 26 RESPONDERS). Stainings were processed and analyzed in the same unit (Pathology Unit, Regina Apostolorum Hospital, Albano Laziale).

Results were described as previously reported for the immunocytochemical analysis [9, 27, 28]. Galectin-3, HBME-1, and CK-19 immunoreactivity was considered positive if >10% of the follicular cells appeared stained. Specifically, immunoreactivity was scored as negative (<10%), focally positive (10–25%), and diffusely positive (>25%). As for Ki-67, immunoreactivity was considered positive if ≥1% of follicular cells appeared stained. All immunostained slides were blindly evaluated by two expert examiners (ST and MDA), and discordant cases were mutually resolved.

### 2.3. Statistical Analysis

The Shapiro–Wilk test was applied to continuous variables to check for distribution normality. Quantitative variables were reported as median with range (min-max) or mean ± standard deviation, depending on data distribution. Categorical variables were reported as absolute frequencies and/or percentages. Continuous variables were compared by the Mann–Whitney test (and the Kruskall–Wallis test) or Student's *t*-test (and ANOVA), depending on data distribution and number of groups [29]. Pearson's Chi-squared test or Fisher exact test, as appropriate, was used to compare absolute frequencies. The diagnostic accuracy of immunocytochemistry markers was measured using sensitivity (Sen), specificity (Spe), the positive predictive value (PPV), and the negative predictive value (NPV). The two markers which correlated with the outcomes, CK-19 and Ki-67, were converted to ordinal data and included in a composite biomarker. We assigned a score of 0 to CK-19 positive, a score of 1 to CK-19 negative staining, a score of 0 to Ki-67 negative, and a score of 1 to Ki-67 positive staining. Then, we calculated this composite biomarker by summating the two scores, and an area under the curve (AUC) was calculated.

Statistical significance was set at *p* < 0.05. All statistical analyses were carried out in the R system for statistical computing (Version 4.0.2; R development Core Team, 2020). The R package MatchIt (1 : 1 matching with the nearest neighbor) was used for the propensity matching process for control group selection.

## 3. Results

### 3.1. Patient Characteristics

Patients were selected with PSM from a multicenter cohort of 406 patients with benign thyroid nodules treated with TA and followed for 5 consecutive years [11]. RESPONDERS (controls) were patients who responded to the first TA and were followed up with no need for further treatment, while NONRESPONDERS (cases) were patients who did not respond to the first TA and were surgically retreated.

We collected the FNAC slides of 50/64 (78%) patients, which included the specimens of 24 NONRESPONDERS and 26 RESPONDERS. All FNAC were performed before TA procedures (before 2014), and according to the current TA guidelines [2], in all of them, the cytology report was diagnostic for benign thyroid nodules (Bethesda II diagnostic category). In NONRESPONDERS, final pathology showed 14 benign nodules, 5 carcinomas (3 papillary and 2 follicular carcinomas), 3 follicular adenomas, and 2 oncocitic adenomas. We excluded from our analysis the five cases of NONRESPONDERS which turned out to be carcinomas, and in the end, we compared 26 RESPONDERS to 19 NONRESPONDERS.

Patient characteristics are reported in Table 1. In both groups, patients were predominantly females in their 50 s; their nodules measured 17 mL (range 12–25) and were mostly solid (that is, with a solid component >90%) and nonfunctioning [2]. Treatment modality was most often laser ablation (84–92% of cases). There were no differences in terms of energy delivered. All patients who were NONRESPONDERS were retreated with surgery; in particular, 79% of them did not show a clinically satisfactory nodule reduction (i.e., nodule volume was not reduced by 50% after 1 year) with symptom persistence, and 74% exhibited a significant nodule regrowth during follow-up (i.e., nodule volume increased >50% as compared to the smallest volume achieved after the procedure). By contrast, in all RESPONDERS, TA achieved technique efficacy and none of the nodules regrew for the five consecutive years after the procedure.

### 3.2. Immunocytochemistry Stainings

FNAC slides were stained for Galectin-3, CK-19, HBME-1, and Ki-67. Groups differed only in terms of CK-19 (Table 2). CK-19 was absent in all the NONRESPONDERS, while 26% (6/23) of the RESPONDERS expressed it (*p*=0.03). There were not differences in terms of Galectin-3, HBME-1, and Ki-67 stainings between the groups. When looking at the nodules expressing Ki67, it was expressed in 1% of follicular cells in 7 samples (3 RESPONDERS and 4 NONRESPONDERS) and 2% in 2 samples (NONRESPONDERS).

### 3.3. Predictive Value of Biomarkers

Table 3 shows the sensitivity, specificity, the positive predictive value, and the negative predictive value of CK-19 and Ki-67 with respect to regrowth and retreatment. The absence of CK-19 was associated with resistance to TA, and consistent with this, CK-19 expression had high sensitivity (100%) and high NPV for regrowth and retreatment, as all patients who had CK-19 expression responded to TA and did not have any regrowth or retreatment. On the other hand, Ki-67 expression was found to be the most specific biomarker for retreatment (88%) with high PPV, as the majority of patients with Ki-67 expression (67%) were retreated.

Then, we considered CK-19 and Ki-67 as two ordinal variables and assigned the score of 0 to CK-19 presence as well as Ki67 absence (response to TA) and the score of 1 to CK-19 absence and Ki-67 presence (no response to TA). This allowed us to perform a ROC analysis and obtain an AUC for this composite biomarker of CK-19 + Ki-67. The AUC for regrowth was 0.61 (95%CI: 0.47-0.75, i.e. almost acceptable discrimination), and the AUC for retreatment was 0.68 (95%CI: 0.55-0.81). The sensitivity, specificity, PPV, NPV, and accuracy of the composite biomarker for regrowth were 23% (5–54%), 85% (66–96), 43% (10–82), 70% (51–84), and 65% (48–79), respectively. The sensitivity, specificity, PPV, NPV, and accuracy of the composite biomarker for retreatment were 29% (10–56), 91% (72–99), 71% (30–97), 64% (45–80), and 65% (48–79), respectively, as shown in Table 4.

## 4. Discussion

Long-term retrospective and prospective studies have demonstrated the influence of size, structure, and vascularization on the clinical outcome of benign thyroid nodules treated with TA [5–10]. Yet, data concerning the effect of the baseline biological characteristics of the nodules on the response to treatment are poor [23, 24]. Our study demonstrates that benign nodules that did not respond successfully to TA and required retreatment did not express CK-19. In other words, the absence of CK-19 was associated with resistance to TA, while the presence of CK-19 was associated with a good response to the treatment. Consistent with this, CK-19 expression had high sensitivity and a high negative predictive value for retreatment and regrowth. When we considered CK-19 absence and Ki-67 presence as two ordinal data and we computed a composite biomarker including both of them, we found that this was a biomarker with a high specificity for retreatment and regrowth and a low rate of false positive results.

The presence of CK-19 was associated with a good response to TA and had a negative predictive value for regrowth and retreatment. CK-19 is a low molecular weight cytokeratin found in basal layers of stratified epithelium and simple epithelia, which is upregulated in neoplastic transformation. It has been shown that papillary thyroid carcinomas exhibit an intense diffuse expression of CK-19, whereas follicular carcinomas show a heterogeneous expression of CK-19, which tends to be absent in follicular adenomas [20, 30, 31]. Likewise, in a study on 18 cases of atypical follicular thyroid nodules characterized by a solid growth pattern, 17/18 cases were negative for CK-19 [30]. Consistent with this finding, Nehal et al. [31] have demonstrated that, in a series of thyroid lesions, CK-19 was expressed in all the papillary thyroid carcinoma specimens, as well as in 87.5% of adenomatous hyperplasia, whereas it was absent in follicular adenomas and follicular carcinomas.

Based on these data, we speculate that benign thyroid nodules expressing CK-19 are likely to be adenomatous hyperplasia rather than follicular (or oncocytic) adenomas. Distinction between these two types of benign thyroid lesions is not always straightforward [32]. Nevertheless, diagnosis of follicular adenoma should be restricted to encapsulated thyroid nodules that show a distinct growth pattern from the surrounding thyroid parenchyma (i.e. microfollicular, or macro-follicular, or trabecular) devoid of degenerative changes [32, 33]. Benign thyroid nodules without these features, showing various forms of degenerative changes and partial or absent encapsulation, should be diagnosed as adenomatous hyperplasia. It has to be noted that based on the 2022 WHO classification of thyroid neoplasms, adenomatous hyperplasia should be now called “thyroid follicular nodular disease” [22]. Nevertheless, according to Nikiforov [34], the biologic difference between adenomatous hyperplasia and follicular adenomas relies on the fact that in the first case nodules are formed by proliferation of multiple cells in a nonclonal process, while in the second case, nodules develop from a single cell in a clonal process.

The observation that the presence of CK-19 is associated with a good response to TA is in line with the results of a recent prospective study [23] comparing laser ablation to radiofrequency ablation for debulking benign nonfunctioning thyroid nodules and showing that volume reduction ranged between 53.2% and 64.3% 6 months after the procedure. In that study, patients underwent core needle biopsy prior to thermal ablation treatment. Immunohistochemistry resulted, as in our study, negative for galectin3 and HBME1, while a few cases exhibited a focal positivity for CK19. There was no association between CK-19 expression and degree of volume reduction. Nodule cellularity was the only baseline predictive variable for technique efficacy (i.e. volume reduction >50% after 1 year).

Importantly, a not negligible percentage of the thyroid nodules not responding to TA resulted at the final pathologic examination as differentiated cancer or neoplasia with uncertain clinical behavior. This finding confirms the opportunity, as recommended by the thyroid TA guidelines [1], of performing a new cytological assessment in the case of a poor response to TA, be it nodule regrowth [1] or lack of technique efficacy [11].

This study has some limitations, including its retrospective design, the small number of cases, and the absence of a final surgical diagnosis in nodules that were successfully treated. Strengths of the study are the selection of groups by the propensity score matching method, the long-term follow-up of successfully treated nodules, the use of a centralized pathology laboratory, and the blinded assessment of the slides. In addition, this is one of the first and few studies trying to identify biological/immunocytochemistry markers of the treatment response in patients undergoing thermal ablations. Future studies on the predictive value of other molecular markers, such as RAS mutations [35] and RET rearrangements, which are present in benign thyroid nodules with a tendency to grow [36–38], can help identify additional biological features predicting resistance to TA.

In conclusion, our data suggest that benign nodules expressing CK-19, which is more frequent in adenomatous hyperplasia, show a clinically satisfactory response to thermal ablation. Further studies are needed to confirm our results and identify further potential markers predictive of the treatment response.

## Figures and Tables

**Table 1:** Patient characteristics.

	Controls (*n* = 26)	Cases (*n* = 19)	*p* value
Age (year)	51.6 (12.6)	49.4 (14.5)	0.36
*F* (%)	23 (88%)	15 (79%)	0.43
Nodule volume (mL)	17.0 (11.8–25.2)	17.4 (14.5–23.0)	0.43
Solid (%)	24 (92%)	15 (79%)	0.25
AFTN (%)	0 (0%)	1 (5%)	0.42
Laser (%)	24 (92%)	16 (84%)	0.63
Energy (J/mL)	521 (89–1777)	494 (375–1515)	0.31
Technique efficacy (%)	26 (100%)	4 (21%)	<0.001
Regrowth (%)	0 (0%)	14 (74%)	<0.001
Retreatment	0 (0%)	19 (100%)	<0.001

Data are expressed as median (min-max). SD is for standard deviation.

**Table 2:** Immunocytochemistry stainings.

	Controls (*n* = 26)	Cases (*n* = 19)	*p* value
GAL-3 (%)	0/23 (0%)	1/17 (5.8%)	0.425
CK-19	6/23 (26%)	0/17 (0%)	0.0295
HBME-1	6/26 (23%)	6/19 (32%)	0.734
Ki67	3/26 (11.5%)	6/19 (32%)	0.137

Data are expressed as % of positive slides.

**Table 3:** Predictive value of biomarkers for response to thermal ablation.

Marker	FNAC samples	Outcome regrowth			Indicators of diagnostic performance % (CI)				
		Yes	No	Total	Sen	Spe	PPV	NPV	AC
CK19	Negative	13	21	34	100 (66–100)	22 (9–42)	38 (22–56)	100 (42–100)	47 (32–64)
	Positive	0	6	6					
	Total	13	27	40					
Ki67	Positive	3	6	9	21 (5–51)	81 (62–92)	33 (7–70)	69 (52–84)	62 (46–76)
	Negative	11	25	36					
	Total	14	31	45					

Marker	FNAC samples	Outcome retreatment			Indicators of diagnostic performance % (CI)				
		Yes	No	Total	Sen	Spe	PPV	NPV	AC

CK19	Negative	17	17	34	100 (73–100)	26 (10–48)	50 (32–68)	100 (42–100)	57 (41–73)
	Positive	0	6	6					
	Total	17	23	40					
Ki67	Positive	6	3	9	32 (13–57)	88 (70–98)	67 (30–93)	64 (46–79)	64 (49-–8)
	Negative	13	23	36					
	Total	19	26	45					

Sen is for sensistivity; Spe is for specificity; PPV is for positive predictive value; NPV is for negative predictive value; AC is for diagnostic accuracy.

**Table 4:** Predictive value of composite biomarker CK-19 + Ki-67 for response to thermal ablation.

Marker	Outcome regrowth			Indicators of diagnostic performance % (CI)				
	Yes	No	Total	Sen	Spe	PPV	NPV	AC
Score 2	3	4	7	23 (5–54)	85 (66–96)	43 (10–82)	70 (51–84)	65 (48–79)
Score 0-1	10	23	33					
Total	13	27	40					

Marker	Outcome retreatment			Indicators of diagnostic performance % (CI)				
	Yes	No	Total	Sen	Spe	PPV	NPV	AC

Score 2	5	2	7	29 (10–56)	91 (72–99)	71 (30–96)	64 (45–80)	65 (48–79)
Score 0-1	12	21	33					
Total	17	23	40					

Sen is for sensistivity; Spe is for specificity; PPV is for positive predictive value; NPV is for negative predictive value; AC is for diagnostic accuracy.

## Data Availability

Some or all datasets generated and/or analyzed during the current study are not publicly available but are available from the corresponding author on reasonable request.
